# Effects of Opiate Dependence Through Different Administration Routes on Pulmonary Inflammation and its Severity

**DOI:** 10.5812/ircmj.7842

**Published:** 2013-10-05

**Authors:** Mohammad Masoomi, Marzieh Tajoddini, Gholamabbas Mohammadi, Reza Malekpoor, Ebrahim Abasi

**Affiliations:** 1 Cardiology Department, Kerman University of Medical Sciences, Kerman, IR Iran

**Keywords:** Opiate Dependence, pulmonary Inflammation, Naloxone

## Abstract

**Background:**

Serious health problems and socioeconomic consequences of the illicit use of opiates have been proved in both developed and developing societies

**Objectives:**

We aimed to evaluate" The effects of opiate addiction through different administration routes on pulmonary inflammation and its severity."

**Materials and Methods:**

Our experiments were performed on eighteen adult male Syrian golden hamsters which were allocated to one of the three groups (n = 6): control group which did not receive opiate; the first study group were administered oral opiate via stomach tube; and another study group were administered inhaled opiate. After four weeks, all hamsters were anesthetized with diethyl ether and their lung tissues were isolated for pathological assessment.

**Results:**

Severe perivascular inflammation was detected in 33.3% of the samples with oral opiate dependence and 20% of the cases addicted to opiate through inhalation. Also, severe peribronchial inflammation was observed in only 20% of the samples addicted to inhaled opiate and was not found in the other groups. No significant differences were found in the severity of perivascular and peribronchial inflammation across the three groups. Although the mean of total inflammation scale in the subjects with oral opiate dependence (3.00 ± 1.79) was numerically higher than that in the inhaled dependence group (1.40 ± 2.60) and the controls (2.25 ± 1.26), this difference was not statistically significant.

**Conclusions:**

Administration did not influence the appearance or severity of pulmonary inflammation in animal models addicted to opiate.

## 1. Background

Serious health problems and socioeconomic consequences of the illicit use of opiates have been proved in both developed and developing societies. Based on the published reports, more than 15 million adults, or about 0.4 percent of the world’s adult population, were estimated to be using these drugs in the early 2000s. In addition, illnesses and deaths due to the illicit opiate use were estimated to account for 0.7 percent of global disability-adjusted life years in 2000 (1). Thus, opiate use is a significant cause of premature mortality and morbidity among adults with a physical and mental heavy burden. Besides its socioeconomical effects, its triggering role on disabling disorders such as cardiovascular disorders has been suggested (2, 3).

Furthermore, regarding suppressive influences of opium addiction on immune system, it have been shown that exogenous opioids tend to suppress the immune system and this negative modulation of immune cells depends on the opioid, its dose and the route of administration (4). For instance, opium can impair granulocyte aggregation and suppresses intracellular killing mechanisms in activated monocytes as well as appears to decrease the immune response by lowering chemotaxis and cell adherence (5). In animal models, opium administration can be associated with alterations in a number of immune parameters such as natural killer cells activity, proliferation of lymphocytes, antibody production and interferon production(6-8). Moreover, most of these immunosuppressive effects can be blocked by opioid antagonists (9). Despite confirmed adverse effects of opium use on immune pathways and cells, its impact on pulmonary function parameters and its immune systems is already questioned. Although some studies implicated opium use as the most likely responsible factor for lowering lung functional capacity in comparison with normal (10), but some others could not indicate its destructive effects on lung volume and compliance (11). Also, effects of different routes of opium administration as a main influencing factor on immune destructive process have not been cleared. Based on an experimental study, we tried to test this hypothesis that the route of opium administration has a major role in the progression of inflammation and its severity in pulmonary system.

## 2. Objectives

We aimed to evaluate" The effects of opiate addiction through different administration routes on pulmonary inflammation and its severity".

## 3. Materials and Methods

Our experiments were performed on 18 adult male Syrian golden hamsters, weighting 90-110 gr. Animals were kept at constant ambient temperature (22°C ± 1°C), under a 12-hour light/dark cycle with free access to food and water for two weeks. Then, hamsters were allocated to one of three groups (n = 6): control group which did not receive opiate; the first study group were administered oral opiate twice a day via stomach tube; and another study group were administered inhaled opiate twice daily. In order to induce oral addiction to opiate, hamsters were addicted to opium through the use of increasing concentrations of opium (0.1, 0.2, 0.3, and 0.4 mg/mL) in their water over three 48-hour periods. For inhaled dependence to opiate, hamsters were transferred to a closed room in order to inhale opiate twice a day in the same periods.

Opium addiction was tested by the injection of Naloxane (2mg/Kg, SC) and the appearance of the withdrawal syndrome. After four weeks, all hamsters were anesthetized with diethyl ether and their lung tissues were isolated for pathological assessment. A random number was assigned to each hematoxylin and eosin-stained lung section from the treatment and control groups. A pathologist blinded to the random numbers evaluated the slides for the degree of inflammation. The degree of peribronchial and perivascular inflammation was evaluated on a subjective scale of 0, 1, 2, and 3, corresponding to the following severity level: 0) no inflammation; 1) occasional cuffing with inflammatory cells; 2) bronchi or vessels surrounded by a thin layer (1-5 cells thick) of inflammatory cells; and 3) bronchi or vessels were surrounded by a thick layer (more than 5 cells thick) of inflammatory cells. The total lung inflammation was defined as the sum of peribronchial and perivascular inflammation scores (12, 13). Results were reported as mean ± standard deviation (SD) for quantitative variables and percentages for categorical variables. The groups were compared using the One-way ANOVA test or Kruskal-Walis H test for continuous variables, and the chi-square test or Fisher's exact test if required for categorical variables. P values of 0.05 or less were considered statistically significant. All the statistical analyses were performed using SPSS version 16.0 (SPSS Inc., Chicago, IL, USA) for Windows.

## 4. Results

**Figure 1.**
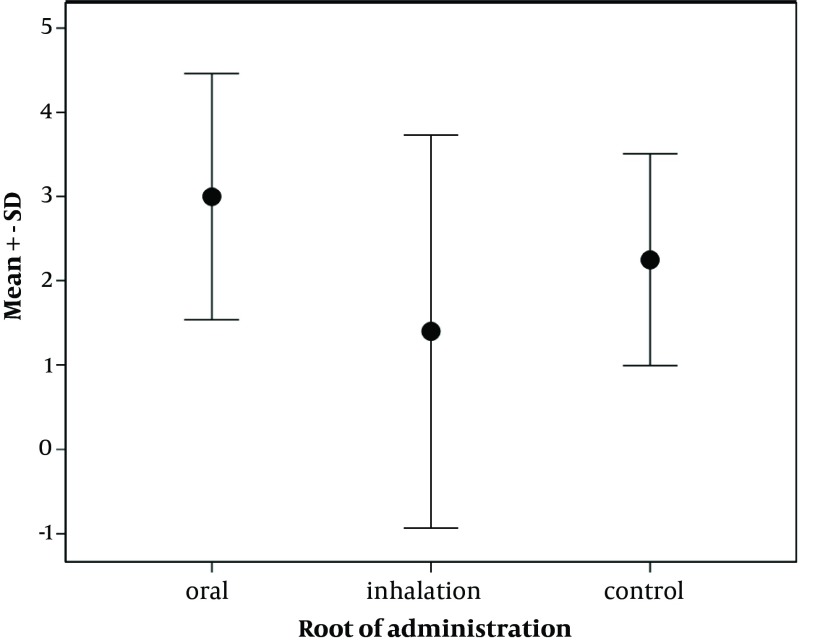
Severity of Perivascular Inflammation in Groups Addicted to Oral and Inhaled Opiate as well as the Control Group

**Figure 2.**
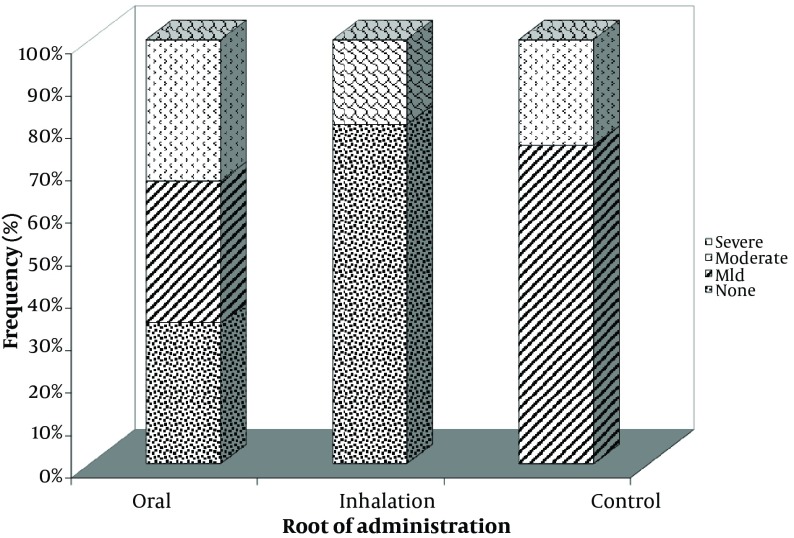
Severity of Peribronchial Inflammation in Groups Addicted to Oral and Inhaled Opiate as well as the Control Group

**Figure 3.**
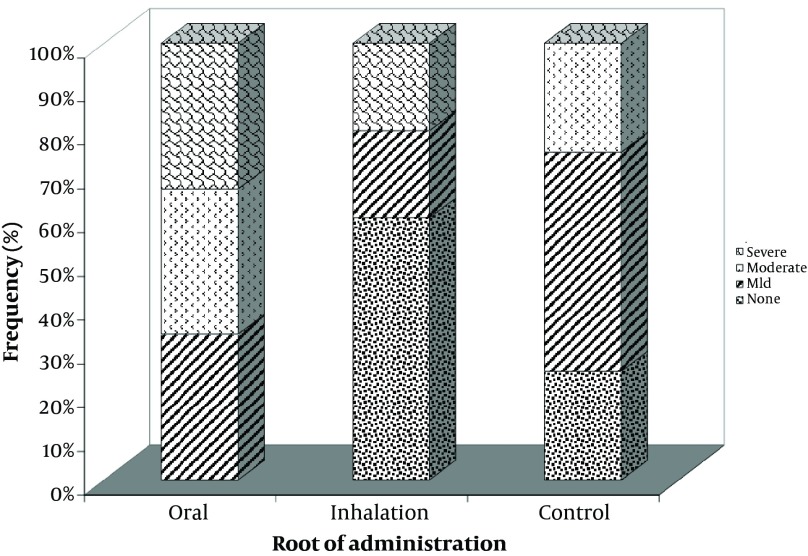
Mean of Total Inflammation Scale in the Groups Addicted to Oral and Inhaled

While preparing the tissues, three isolated samples in the control group were destructed and thus were excluded from the study. Severe perivascular inflammation was detected in 33.3% of the samples with oral opiate dependence and 20% of the cases addicted to opiate through inhalation, while, severe perivascular inflammation was not observed in the control group (Figure 1). Besides, this type of inflammation was not found in 60% of hamsters in the groups administered inhaled opiate and 25% of those in the control group. No significant difference was found in the severity of perivascular inflammation across the three groups (P = 0.302). Regarding the appearance of peribronchial inflammation and its level (Figure 2), severe peribronchial inflammation was observed only in 20% of the subjects addicted to inhaled opiate and was not found in other groups, whereas mild to moderate peribronchial inflammation was revealed in 66.6% of the oral addicted group and in 100% of the control group. No difference was also found in the severity of peribronchial inflammation between the three study groups (P = 0.088). Although the mean of total inflammation scale in the subjects with oral opiate dependence (3.00 ± 1.79) was numerically higher than that in the inhaled dependence group (1.40 ± 2.60) and the controls (2.25 ± 1.26), this difference was not statistically significant (P = 0.442) (Figure 3). 

## 5. Discussion

Effects of opiates on inflammatory processes have been considered in several experimental systems. Most data were collected during experiments on the elicited paw inflammation in rats and mice (14-19). However, only a few investigations were performed on experimental pulmonary inflammation. The current study came to address the effects of opiates through different administration routes on inflammation and its severity. Our survey had two important findings. First, we showed that opiates in our selected dosage had no significant effects on the appearance of the inflammation as well as on its pathological severity. In fact, the level of pulmonary perivascular and peribronchial inflammation was similar across the study and the control groups. Second, we revealed that the route of administration had no critical role in the determination of the inflammation severity in the pulmonary system. Previously, it was emphasized the triggering role of opiates on inflammatory processes in vital organs. But nowadays, the obtained results has been suggesting a specific modulatory anti-inflammatory role of these substances in the acute inflammatory responses of the animal models (19, 20).

Even, recent studies suggest that peripheral morphine, the most applied opium, may be useful for the clinical management of acute inflammatory disorders (21). Moreover, significant increased concentrations of endogenous opioids in the serum of patients with systemic infection, particularly secreted from neutrophils during sepsis can emphasize the protective role of opiates in inflammatory processes (22). It is believed that low-dose opium might decrease the number of inflammatory leukocytes by inhibiting their migration from hemopoietic sites. The inhibitory effects of morphine on both the cell number and chemoattractant level are completely reversed by the naltrexone pretreatment, which implicates the involvement of opioid receptors (23). Opiates can also interact with interleukins and may act as signaling molecules between immunologically active cells. On the other hand, exogenous opioids tend to suppress the immune system; information on infections in opioid addicts suggest that this has clinical significance (24).However, it should be noticed that the administration of opiates with higher dosages might potentially lead to life-threatening immunosuppression. In addition, besides its psychological and social side effects, opium misuse can adversely affect other vital organs, including cardiovascular or cerebrovascular system that has been previously demonstrated. Totally, our study did not confirm the protective or triggering role of opiate dependence through different administration routes and also did not demonstrate its influence on the severity of pulmonary inflammation. However, more studies on animal models are recommended to confirm current findings.
